# Role of metabolic equivalent between calcium intake and vertebral fractures: a cross-sectional study of NHANES 2013–2014

**DOI:** 10.1186/s12877-022-03666-4

**Published:** 2022-12-20

**Authors:** Hecheng Yu, Zhiqiang Tao, Xiaoming Luo, Ben Huang, Longdian Zhou

**Affiliations:** 1 Department of Rehabilitation Medicine, Shanghai Tenth People’s Hospital, Tongji University School of Medicine, Shanghai, 200040 People’s Republic of China; 2 Department of Spine Surgery, Nanchang Hongdu Hospital of TCM, No. 1399 Diezihu Road, Honggutan New District, Nanchang, 330008 Jiangxi People’s Republic of China; 3 Department of Physical Therapy, Shanghai YangZhi Rehabilitation Hospital (Shanghai Sunshine Rehabilitation Center), Tongji University School of Medicine, Shanghai, 201600 People’s Republic of China

**Keywords:** Vertebral fracture, Metabolic equivalent, Calcium intake, NHANES

## Abstract

**Background:**

This study was to analyze the association of calcium intake and metabolic equivalent (MET) with vertebral fractures, and to explore the role of MET between calcium intake and vertebral fractures.

**Method:**

This cross-sectional study used data from the National Health and Nutrition Examination Surveys (NHANES) 2013–2014. The study involved individuals aged ≥ 50 years old with complete information on vertebral fracture, calcium intake, and physical activity. Vertebral fracture assessment is obtained using dual-energy x-ray absorptiometry to perform a lateral scan of the thoracolumbar spine. Calcium intake included total nutrient intake and total dietary supplements. The total MET is the sum of the METs for each activity (Vigorous/ moderate work-related activities, walking or bicycling for transportation and vigorous/ moderate recreational activities). Univariate and multivariate logistic regression analyses were utilized to investigate the effect of calcium intake, MET, and their combined effect on vertebral fracture.

**Results:**

A total of 766 participants were included in the analysis, and 54 participants had vertebral fractures. The median calcium intake and MET were 8.43 mcg and 280.00, respectively. Multivariate results showed that neither calcium intake nor MET as continuous or categorical variables was significantly associated with vertebral fractures. MET < 160 and calcium intake ≥ 670 mg group was associated with the decreased risks of vertebral fracture [odds ratio (OR) = 0.47, 95% confidence interval (CI): 0.26–0.83, *P* = 0.032] after adjusting for age, race, energy, total femur bone mineral density (BMD), and femoral neck BMD. In the group of MET < 160, increased calcium intake was associated with a reduced risk of vertebral fracture, with a decreased OR value. In the group of MET ≥ 160, increased calcium intake was associated with an increased risk of vertebral fracture, with an increased OR value.

**Conclusion:**

The combination of MET < 160 and calcium intake ≥ 670 mg was associated with decreased risks of vertebral fractures. There may be an interaction between calcium intake and MET on vertebral fracture risk.

**Supplementary Information:**

The online version contains supplementary material available at 10.1186/s12877-022-03666-4.

## Background

Vertebral fractures are a common type of fracture in people over 50 years old, they tended to occur in postmenopausal women and are associated with osteoporosis [1, 2]. Vertebral fractures can lead to acute and chronic pain, height loss, kyphosis, impaired activities of daily living, psychological distress, decreased quality of life, and reduced life expectancy [3–5]. Although the definition of vertebral fractures may vary between studies, it has consistently been found to affect at least 20% of people older than 50 years of age [4]. The incidence of vertebral fractures reached more than 50% with age, especially in postmenopausal women [6, 7]. There is no consensus on the optimal treatment of vertebral fractures, with conservative treatment options including oral analgesics, rehabilitative exercises, spinal orthoses, and multimodal therapy [8], while more invasive treatment options such as kyphoplasty and vertebroplasty have been shown to be superior to conservative treatment [1, 9–11]. Therefore, early implementation of the appropriate intervention is of great significance to reduce the risk of vertebral fractures, improve the quality of life and prolong life.

Nutritional intake and exercise are important for preventing osteoporosis and avoiding fractures caused by osteoporosis [12–14]. Calcium is an important element in human physiology and plays a key role in muscle contraction, bone strength, nerve impulses and transmission [15]. Inadequate calcium intake results in poor peak bone mass and low bone mineralization, which may lead to osteoporosis and fractures [12]. In a study based on a Chinese population, higher dietary calcium intake was associated with a lower risk of vertebral fracture in women, but no such association was found in men [16]. Exercise can improve or maintain bone mass at all ages, and the mechanical stress generated during exercise causes certain deformation of bone tissue, thereby stimulating osteoclasts and osteoblasts and enhancing bone strength [17]. A study based on the Swedish population suggested that self-reported recreational activities intensity and frequency were associated with fracture risk, and moderate and high-frequency physical activity were significantly associated with a reduction in future fracture risk [18].

In children and adolescents, regular physical exercise combined with high levels of calcium intake is beneficial to bone health in young people [19]. Physical exercise could moderate the adverse effects of low calcium intake on bone loss in perimenopausal and postmenopausal women [20]. These studies suggested that exercise and calcium intake had certain effects on bone health. However, few studies on fracture risk have used uniform quantitative assessment criteria to evaluate exercise levels. In addition, more attention has been paid to the effect of calcium and other multi-nutrient supplements on fracture risk, and whether the combination of nutrition and exercise benefit the population remains to be further confirmed. Compared with other types of fractures, vertebral fractures lack specific manifestations in the early stage, and are mostly found after imaging-assisted diagnosis [21], which clacked a large number of epidemiological data.

Therefore, this study used a standardized method to assess the level of physical activity, namely metabolic equivalent (MET), to analyze the association of calcium intake and MET with vertebral fractures, and to explore the role of MET between calcium intake and vertebral fracture risk, providing guidance on population intervention and management.

## Methods

### Study population

We analyzed the data from the National Health and Nutrition Examination Surveys (NHANES) 2013–2014, a representative cross-sectional survey of all non-institutionalized civilian populations in the United States (https://www.cdc.gov/nchs/nhanes/index.htm). Data collection and analysis for this study were performed in strict accordance with the tutorial and analysis guidelines for the NHANES database. The study involved individuals (1) aged ≥ 50 years old, (2) with the assessment of vertebral fractures, (3) with complete calcium intake data, and (4) with information on physical activity. Excluded criteria were (1) premenopausal and perimenopausal women, (2) participants with missing key covariates such as bone mineral density (BMD), family income to poverty ratio, body mass index (BMI), smoking status, and education level, and (3) participants combined with malignant tumors. Women were categorized as postmenopausal if they answered “menopause” to “What is the reason that you have not had a period in the past 12 months?”.

The requirement of ethical approval for this study was waived by the Institutional Review Board of Nanchang Hongdu Hospital of TCM, because the data was accessed from NHANES (a publicly available database). All individuals provided written informed consent before participating in the study. All methods were carried out in accordance with relevant guidelines and regulations (Declaration of Helsinki) [22].

### Vertebral fracture

Vertebral fracture assessment was obtained using dual-energy x-ray absorptiometry (DXA) to perform a lateral scan of the thoracolumbar spine [23]. All scans were analyzed by Optasia Spinalizer software using Genant’s semiquantitative (SQ) technique [24]. Grade 0 indicates normal, Grade 1 indicates mild vertebral fracture, Grade 2 indicates moderate vertebral fracture, Grade 3 indicates severe vertebral fracture, and Grade 1 or higher are diagnosed as vertebral fractures [25].

### Calcium intake

Calcium intake included total nutrient intake and total dietary supplements. All participants were eligible to participate in two 24-h dietary recall interviews. The first dietary recall interview was collected in person at the Mobile Examination Center (MEC) and the second interview was collected by telephone 3 to 10 days later [25, 26]. Calcium intake was treated as a continuous and categorical variable. Categorical variables were categorized by the following quartiles, with a cutoff value of 670 mg.

#### MET

Vigorous work-related activities and moderate work-related activities were assessed to question “How much time do you spend doing vigorous-intensity/ moderate-intensity activities at work on a typical day?” (PAD615 and PAD630). The score of PAD615 and PAD630 were 8.0 and 4.0, respectively. Walking or bicycling for transportation was assessed to the question “How much time do you spend walking or bicycling for travel on a typical day?” (PAD645). The score of PAD645 was 4.0. Vigorous recreational activities and moderate recreational activities were assessed according to question “How much time do you spend doing vigorous-intensity/ moderate-intensity sports, fitness or recreational activities on a typical day?” (PAD660 and PAD675). The score of PAD660 and PAD675 were 8.0 and 4.0, respectively. The MET of each activity is equal to the exercise time of each activity multiplied by the score of the corresponding question. The total MET is the sum of the METs for each activity and represents a whole day of activity. MET was treated as continuous and categorical variables. Categorical variables were categorized by the following quartiles, with a cutoff value of 160.

### Data collection

Demographic variables such as age, gender (male or female), race [White or others (Mexican American, other Hispanic, Non-Hispanic Black, other races)], education level [less than 9th grade, 9-11th grade (includes 12th grade with no diploma), high school graduate/ general educational development (GED) or equivalent, some college or AA degree, or college graduate or above], and family income to poverty ratio were collected. Examination data including height (cm), weight (kg), waist circumference (cm), BMI (kg/m^2^) were also collected. Smoking was defined as someone who smoked at least one hundred cigarettes in life. Drinking was defined as participants drinking at least 12 alcoholic drinks per year. Diabetes was determined based on laboratory tests, self-reports, and medication history. Diabetes laboratory diagnostic criteria are fasting blood glucose ≥ 7.0 mmol/L or glycosylated hemoglobin (HbAlc) ≥ 6.5%. Drug treatment information including lipid-lowering agents, anti-osteoporosis therapy, and glucocorticoids was also collected through database records. Participants’ parents ever fractured a hip was asked through the questionnaire. BMD was measured using DXA. BMD at the femur neck and total femur was used to calculate the T-score [respondent’s BMD-reference group mean BMD)/reference group standard deviation (SD)]. The reference group for the femoral neck consisted of non-Hispanic White women aged 20–29 years from NHANES III [27]. Measured osteopenia was defined as femur neck or total femur BMD T-score ≤  − 1.

### Statistical analysis

Three weighted variables were used in this study. The MEC exam weight (wtmec2yr) was used for weighting. The masked variance unit pseudo-stratum was sdmvstra, and the masked variance unit pseudo-primary sampling units (PSUs) was sdmvpsu. The study population was divided into the vertebral fracture and non-vertebral fracture groups. Normal data were described as mean ± SD, and a comparison between groups was performed by t-test. Non-normal data were described as a median and interquartile range [M (Q1, Q3)], and a comparison between groups was performed by the Mann–Whitney U rank sum test. Enumeration data were described by the number of cases and the constituent ratio [n (%)], and comparison between groups was performed by the chi-square test. The missing data was deleted, and a sensitivity analysis was conducted. The ratio before and after deletion of missing values was 84.27%. Variables with significant differences in comparisons between groups (*P* < 0.05), and energy as covariates. Although energy was not significant in the comparison, energy intake was a variable that had to be considered in food intake [28]. Age, race, femoral neck BMD, femoral neck BMD, and energy were covariates. Univariate and multivariate logistic regression analyses were performed with calcium intake and MET, and the combination of calcium intake and MET as independent variables and vertebral fracture as dependent variables. Curves were drawn to explore the effect of different levels of calcium intake with continuously varying MET on vertebral fracture and the effect of different levels of MET with continuously varying calcium intake.

## Results

### Characteristics of the study population

**Fig. 1:**
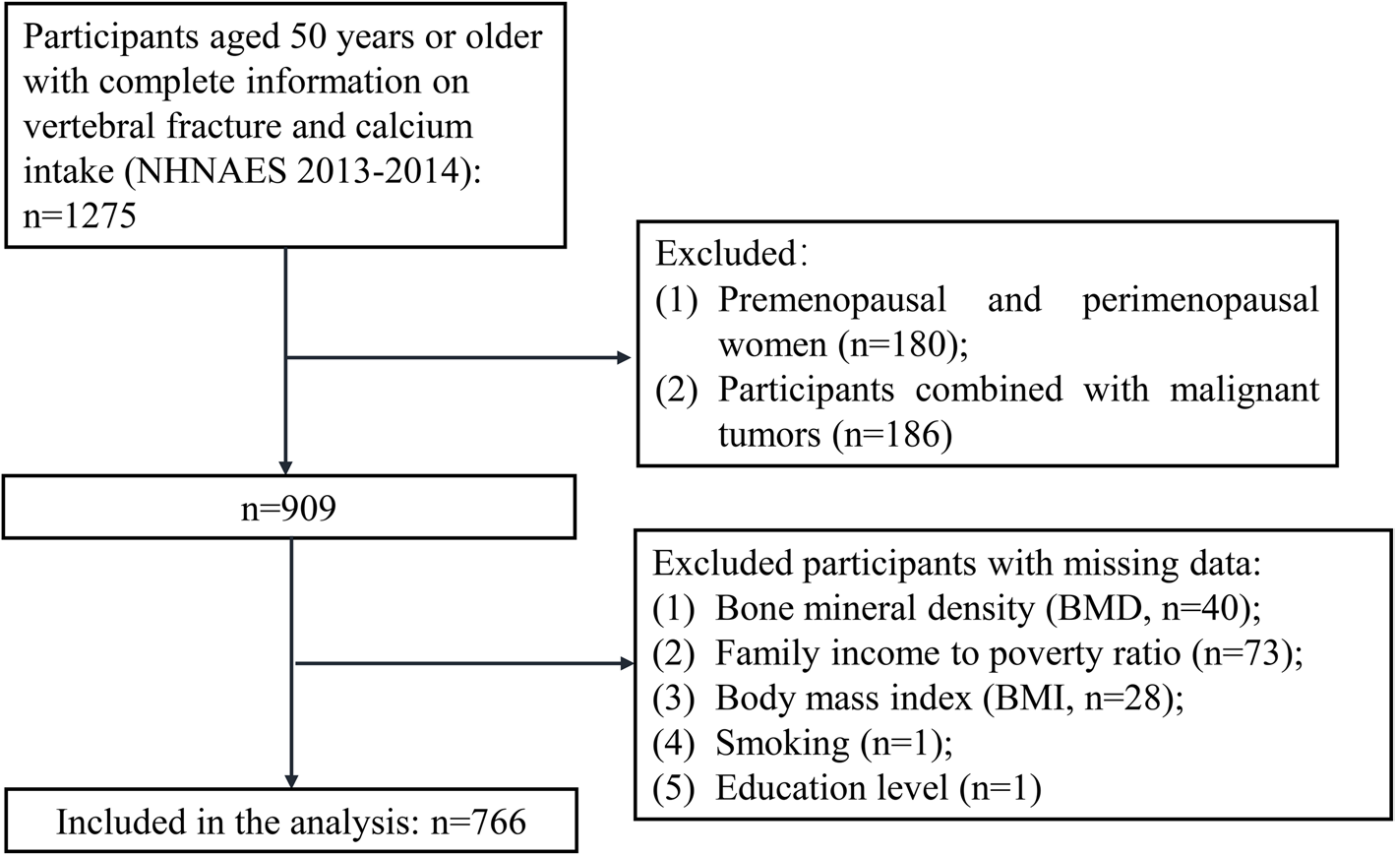
Flowchart of study population screening

A total of 1,275 participants (≥ 50 years) with complete vertebral fracture and calcium intake information were extracted from NHNAES 2013–2014. After excluding 180 premenopausal and perimenopausal women, 186 participants with comorbid malignancies, and 143 participants with missing key covariates, 766 participants were included in the analysis (Fig. 1). Of these 766 participants, 54 had vertebral fracture. Table 1 demonstrates the characteristics of the study population. The mean ± SD of age was 63.10 ± 8.23 years. There were 303 (39.56%) females and 339 (44.26%) whites. The majority of participants’ education level was college graduate or above [233 (30.42%)], followed by some college degree [208 (27.15%)], and high school graduate/GED or equivalent [161 (21.02%)]. Overall, the median of total calcium intake was 8.43 mcg, and the median MET was 280.00. For process of the missing values, no significant differences were found between before and after deleting the missing value (Supplementary Table 1).

**Table 1:** Characteristics of the study population and comparison between vertebral fracture and non-vertebral fracture groups

Variables	Total (*n* = 766)	Groups		Statistics	*P*
		Non-Vertebral fracture (*n* = 712)	Vertebral fracture (*n* = 54)		
Age, years, Mean ± SD	63.10 ± 8.23	62.89 ± 8.10	65.89 ± 9.50	t = -2.59	0.010
Gender, n (%)				χ^2^ = 0.46	0.496
Male	463 (60.44)	428 (60.11)	35 (64.81)		
Female	303 (39.56)	284 (39.89)	19 (35.19)		
Race, n (%)				χ^2^ = 11.83	< 0.001
White	339 (44.26)	303 (42.56)	36 (66.67)		
Others (Mexican American, other Hispanic, non-Hispanic Black, and other races)	427 (55.74)	409 (57.44)	18 (33.33)		
Education level, n (%)				χ^2^ = 1.77	0.777
Less than 9th grade	65 (8.49)	62 (8.71)	3 (5.56)		
9-11th grade (includes 12th grade with no diploma)	99 (12.92)	94 (13.20)	5 (9.26)		
High school graduate/GED or equivalent	161 (21.02)	149 (20.93)	12 (22.22)		
Some college or AA degree	208 (27.15)	191 (26.83)	17 (31.48)		
College graduate or above	233 (30.42)	216 (30.34)	17 (31.48)		
Family income to poverty ratio, M (Q_1_, Q_3_)	2.46 (1.21, 5.00)	2.55 (1.22, 5.00)	1.99 (1.02, 3.57)	Z = -1.88	0.060
Height, cm, Mean ± SD	168.02 ± 9.81	168.07 ± 9.87	167.36 ± 9.05	t = 0.51	0.610
Weight, kg, Mean ± SD	79.13 ± 18.20	79.18 ± 18.36	78.47 ± 16.01	t = 0.28	0.781
Waist circumference, cm, Mean ± SD	99.31 ± 13.95	99.20 ± 13.93	100.71 ± 14.24	t = -0.77	0.443
BMI, kg/m^2^, Mean ± SD	27.90 ± 5.40	27.89 ± 5.41	28.02 ± 5.37	t = -0.17	0.864
Smoking, n (%)				χ^2^ = 0.58	0.445
Yes	373 (48.69)	344 (48.31)	29 (53.70)		
No	393 (51.31)	368 (51.69)	25 (46.30)		
Drinking, n (%)				χ^2^ = 1.89	0.169
Yes	579 (75.59)	534 (75.00)	45 (83.33)		
No	187 (24.41)	178 (25.00)	9 (16.67)		
Diabetes, n (%)				χ^2^ = 0.24	0.628
No	588 (76.76)	548 (76.97)	40 (74.07)		
Yes	178 (23.24)	164 (23.03)	14 (25.93)		
Mother ever fracture a hip, n (%)				-	0.359
Yes	40 (5.22)	36 (5.06)	4 (7.41)		
No	699 (91.25)	652 (91.57)	47 (87.04)		
Unknown	27 (3.52)	24 (3.37)	3 (5.56)		
Father ever fracture a hip, n (%)				-	0.142
Yes	22 (2.87)	21 (2.95)	1 (1.85)		
No	704 (91.91)	657 (92.28)	47 (87.04)		
Unknown	40 (5.22)	34 (4.78)	6 (11.11)		
Broken or fractured hip, n (%)				-	0.521
Yes	10 (1.31)	9 (1.26)	1 (1.85)		
No	756 (98.69)	703 (98.74)	53 (98.15)		
Lipid-lowering agents, n (%)				χ^2^ = 0.02	0.891
No	503 (65.67)	468 (65.73)	35 (64.81)		
Yes	263 (34.33)	244 (34.27)	19 (35.19)		
Anti-osteoporosis therapy, n (%)				χ^2^ = 0.85	0.358
No	755 (98.56)	701 (98.46)	54 (100.00)		
Yes	11 (1.44)	11 (1.54)	0 (0.00)		
Glucocorticoids, n (%)				-	1.000
No	751 (98.04)	698 (98.03)	53 (98.15)		
Yes	15 (1.96)	14 (1.97)	1 (1.85)		
Total femur BMD, gm/cm2, Mean ± SD	0.95 ± 0.15	0.95 ± 0.15	0.89 ± 0.13	t = 2.61	0.009
Femoral neck BMD, gm/cm2, Mean ± SD	0.77 ± 0.14	0.78 ± 0.14	0.73 ± 0.11	t = 2.92	0.005
T-score of total femoral, M (Q_1_, Q_3_)	-0.03 (-0.72, 0.63)	-0.01 (-0.69, 0.65)	-0.24 (-0.91, 0.26)	Z = -2.48	0.013
T-score of femoral neck, M (Q_1_, Q_3_)	-0.08 (-0.71, 0.61)	-0.06 (-0.71, 0.64)	-0.31 (-0.91, 0.26)	Z = -2.19	0.029
Osteopenia, n (%)				χ^2^ = 1.49	0.223
Yes	150 (19.58)	136 (19.10)	14 (25.93)		
No	616 (80.42)	576 (80.90)	40 (74.07)		
Energy, kcal, M (Q_1_, Q_3_)	1867.75 (1440.00, 2410.00)	1861.75 (1428.50, 2406.00)	1990.75 (1560.00, 2491.00)	Z = 1.03	0.304
Vitamin D, mcg, M (Q_1_, Q_3_)	8.43 (3.00, 22.45)	8.58 (2.95, 23.03)	7.48 (3.55, 21.25)	Z = -0.11	0.909
Total calcium intake, mg, M (Q_1_, Q_3_)	981.00 (670.50, 1436.00)	979.75 (669.75, 1425.50)	995.75 (731.00, 1613.50)	Z = 1.15	0.252
MET, M (Q_1_, Q_3_)	280.00 (160.00, 480.00)	244.00 (160.00, 480.00)	450.00 (180.00, 640.00)	Z = 2.05	0.041

*SD* standard deviation, *M* median, *Q*_1_ 1st quartile, *Q*_3_ 3rd quartile, *BMI* body mass index, *MET* metabolic equivalent, *BMD* bone mineral density

### Comparison of vertebral fracture and non-vertebral fracture groups

Table 1 shows the significant differences in age (*P* = 0.001), race (*P* < 0.001), total femur BMD (*P* = 0.013), femoral neck BMD (*P* = 0.029), and MET (*P* = 0.041) between vertebral fracture and non-vertebral fracture groups. The age (65.89 ± 9.50 vs. 62.89 ± 8.10 years old) and MET [450.00 (180.00, 640.00) vs. 244.00 (160.00, 480.00)] of the vertebral fracture group were significantly higher than those of the non-vertebral fracture group. The total femur BMD [-0.24 (-0.91, 0.26) vs. -0.01 (-0.69, 0.65) gm/cm^2^] and femoral neck BMD [-0.31 (-0.91, 0.26) vs. -0.06 (-0.71, 0.64) gm/cm^2^] of vertebral fracture group was significantly lower than that of the non-fracture group (Table 1).

### Association of calcium intake, MET, and their combination on vertebral fracture

Univariate and multivariate results showed that neither calcium intake nor MET as continuous or categorical variables was significantly associated with vertebral fractures (Table 2). Table 3 shows the association of calcium intake and MET combination on vertebral fracture. MET < 160 and calcium intake ≥ 670 mg was associated with the decreased risk of vertebral fracture [odds ratio (OR) = 0.47, 95% confidence interval (CI): 0.26–0.83, *P* = 0.032] after adjusting for age, race, energy, total femur BMD, and femoral neck BMD.

**Table 2:** Independent association of calcium intake and MET on vertebral fracture

	Model 1		Model 2	
	OR (95%CI)	*P*	OR (95%CI)	*P*
Calcium intake	1.24 (0.92–1.68)	0.177	1.12 (0.85–1.47)	0.450
Level of calcium intake				
< 670 mg	Ref		Ref	
≥ 670 mg	1.25 (0.57–2.77)	0.589	1.08 (0.49–2.38)	0.852
MET	1.24 (0.92–1.68)	0.185	1.28 (0.93–1.76)	0.162
Level of MET				
< 160	Ref		Ref	
≥ 160	1.56 (0.80–3.03)	0.210	1.71 (0.88–3.32)	0.151

Model 1 was a univariable model; Model 2 adjusted for age, race, energy, total femur bone mineral density (BMD), and femoral neck BMD

*OR* odds ratio, *CI* confidence interval, *Ref* reference, *MET* metabolic equivalent

**Table 3:** Association of calcium intake and MET combination on vertebral fracture

	Model 1		Model 2	
	OR (95%CI)	*P*	OR (95%CI)	*P*
Calcium intake and MET				
MET ≥ 160 & Calcium intake ≥ 670 mg	Ref		Ref	
MET < 160 & Calcium intake ≥ 670 mg	0.48 (0.27–0.87)	0.034	0.47 (0.26–0.83)	0.032
MET < 160 & Calcium intake < 670 mg	0.89 (0.21–3.66)	0.871	0.94 (0.22–4.04)	0.938
MET ≥ 160 & Calcium intake < 670 mg	0.64 (0.25–1.62)	0.366	0.72 (0.28–1.83)	0.509

Model 1 was a univariable model;

Model 2 adjusted for age, race, energy, total femur bone mineral density (BMD), and femoral neck BMD

*OR* odds ratio, *CI* confidence interval, *Ref* reference, *MET* metabolic equivalent

### Different levels of calcium intake and MET on vertebral fracture

**Fig. 2:**
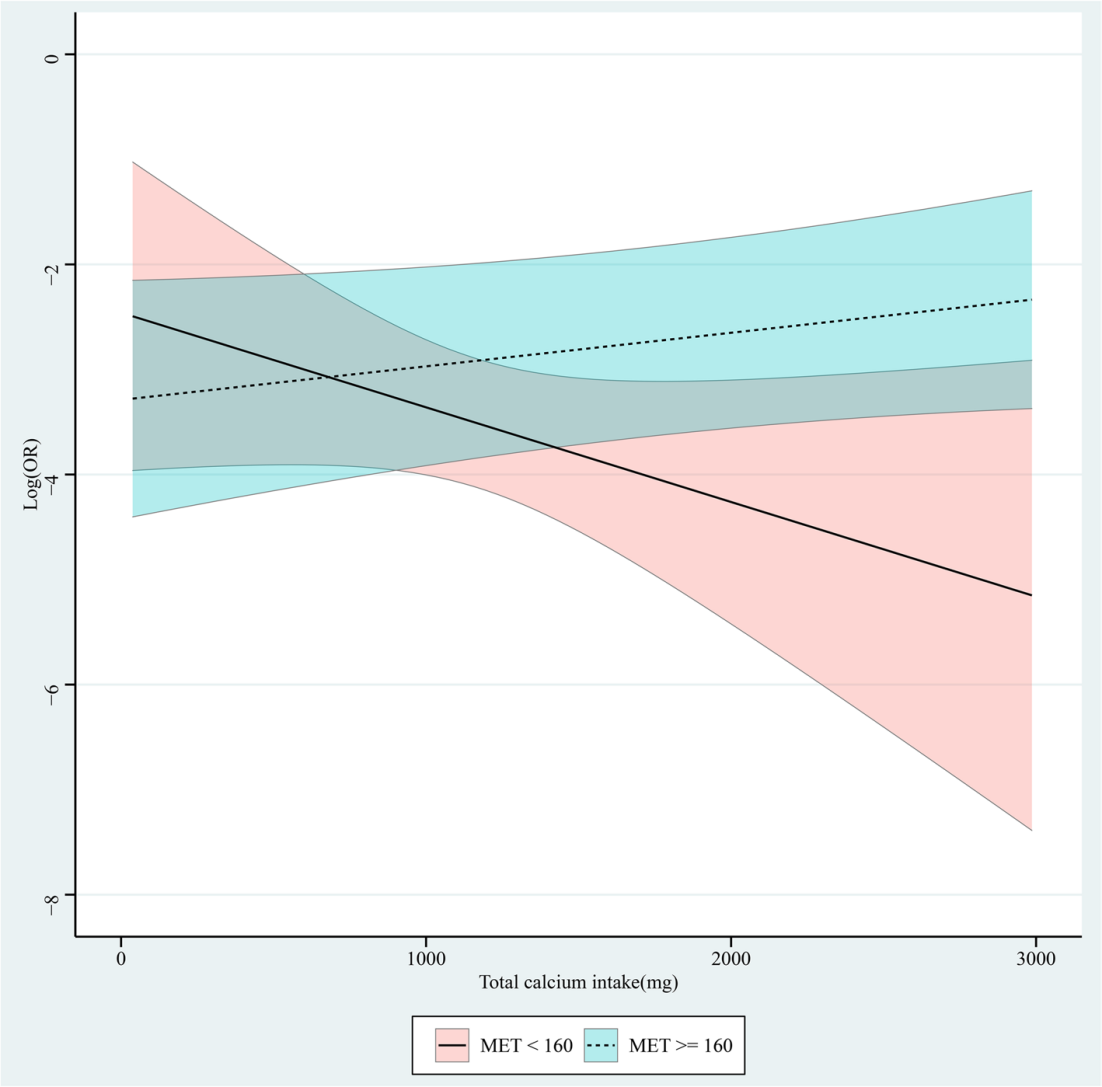
Role of continuous changes in metabolic equivalent (MET) at different levels of calcium intake

**Fig. 3:**
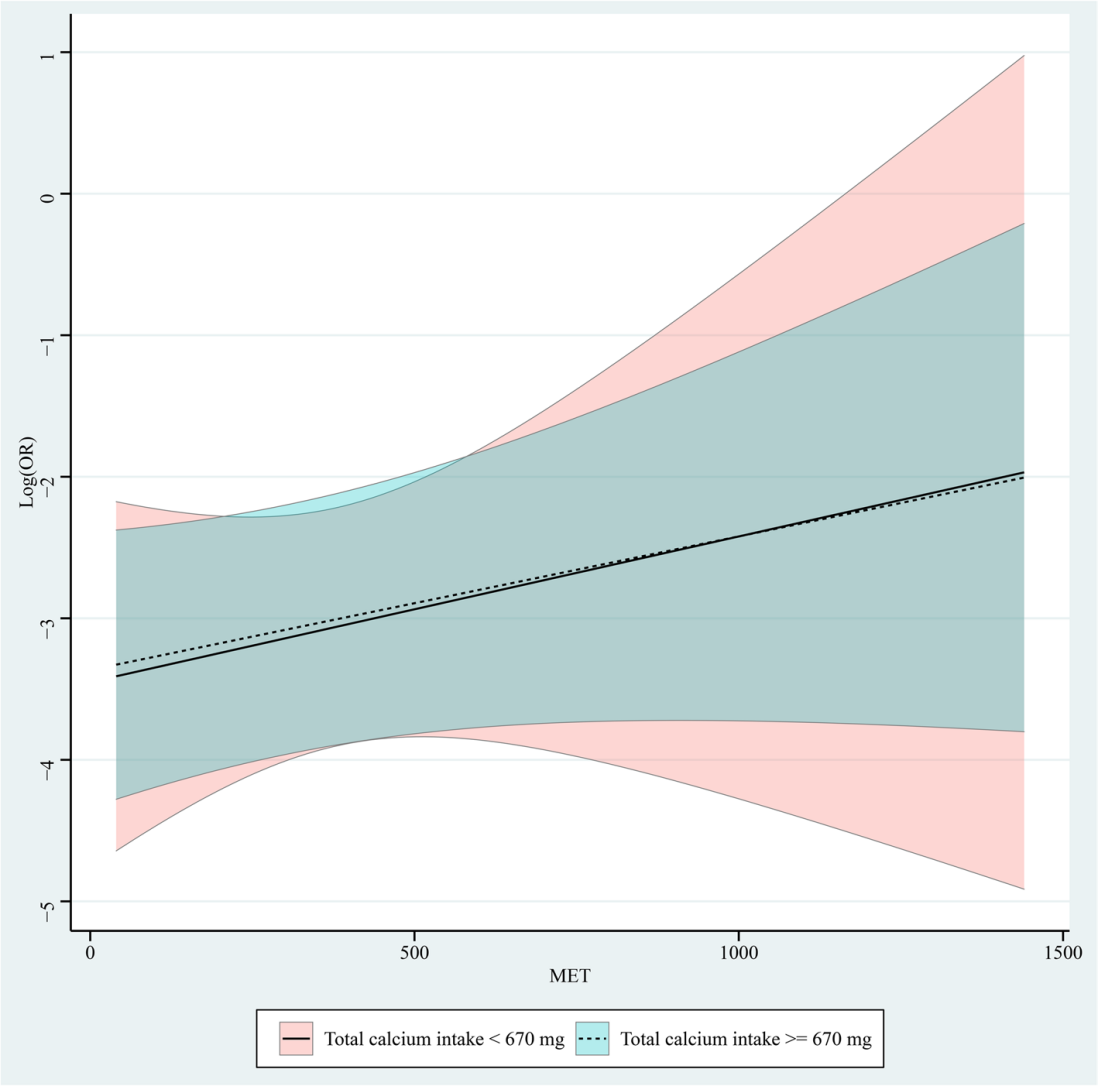
Role of continuous changes in calcium intake at different levels of metabolic equivalent (MET)

Role of continuous changes in MET at different levels of calcium intake was shown in Fig. 2. In the group with MET < 160, increased calcium intake was associated with a reduced risk of vertebral fracture, with a decreased OR value. In the group with MET ≥ 160, increased calcium intake was associated with an increased risk of vertebral fracture, with an increased OR value. Figure 3 is the role of continuous changes in calcium intake at different levels of MET. In groups of calcium intake < 670 mg and ≥ 670 mg, increased MET was associated with an increased risk of vertebral fracture, with an increased OR value.

## Discussion

In this study, we found that calcium intake and MET were not significantly associated with vertebral fractures, respectively. MET < 160 and calcium intake ≥ 670 mg was associated with the decreased risks of vertebral fracture adjusting for age, race, energy, total femur BMD, and femoral neck BMD. In the group of MET < 160, increased calcium intake was associated with a reduced risk of vertebral fracture, with a decreased OR value. In the groups of MET ≥ 160, calcium intake < 670 mg and ≥ 670 mg, increased calcium intake or MET was associated with an increased risk of vertebral fracture, with an increased OR value.

Decreased bone mass and increased bone fragility are the main features of osteoporosis, even slightly during the period of external force could also lead to the occurrence of fractures [29]. Existing studies have confirmed that weakened bone strength was one of the fundamental causes of vertebral fractures [30, 31]. Therefore, this index was often used as a marker to predict fracture risk in clinical practice. A study by Tai et al. showed a slight increase in BMD in the lumbar spine, total hip, femoral neck, and whole body by increasing dietary sources of calcium or taking calcium supplements [32]. We found that calcium intake was not significantly associated with vertebral fractures. The results of several meta-analyses and systematic reviews suggested that dietary calcium intake or supplementation may not be associated with fracture risk in people older than 50 years of age [33, 34], which was consistent with our results. A study has shown that a daily intake of calcium supplements can help prevent fractures, but it is only one essential element of fracture prevention [35]. Efforts to prevent bone loss and osteoporosis should include maintaining a healthy lifestyle, optimal intake of calcium and vitamin D, proper nutrition, adequate sun exposure, exercise, and more [35].

Exercise affects bone strength and quality at all ages. Regular physical activity promotes bone mass gain and bone geometry optimization in childhood and adolescence [30], contributes to bone mass maintenance in adulthood, and reduces bone loss and strength in old age, preventing osteoporosis and sexual fracture in older adults [31]. Physical activity produces external (ground reaction and inertia) and internal (skeletal muscle) forces on the bone, and these forces cause very small deformations in the bone tissue. When an unusual strain is felt, the bone cells initiate an adaptive response through the action of osteoclasts, which resorb bone tissue, and osteoblasts, which produce new bone tissue [36, 37]. Fractures occur when the force exerted on the bone exceeds its strength. Although MET was not found to be significantly associated with vertebral fractures in our study, the combination of MET < 160 and calcium intake ≥ 670 mg was associated with the decreased risks of vertebral fracture after adjusting for confounders. In addition, increased calcium intake or increased MET was associated with an increased risk of vertebral fracture in the group of MET ≥ 160, calcium intake < 670 mg and ≥ 670 mg. It is worth noting that the OR values decreased with increasing calcium intake only in the group of MET < 160. We speculated that MET may play a role in the regulation of calcium intake and vertebral fractures and recommend that exercise with a MET of 160 or more should not be performed in persons over 50 years of age. Studies have shown that dietary calcium and calcium supplements can also have positive effects on bone health, such as postmenopausal women with osteoporosis should receive 1500 mg of calcium per day [38]. Exercise and physical therapy can also strengthen bones to prevent fractures. Strengthening the spinal extensor muscles can increase bone density and reduce the risk of vertebral fractures [39]. Both adequate nutrient intake and physical activity decrease the likelihood of falls and fractures [40]. High-intensity exercise is not recommended for people over 50 based on our results. High-intensity and high-volume training can lead to menstrual dysfunction, reduced bone mineral density, and delayed bone growth [41]. The National Osteoporosis Foundation (NOF) recommended that people who have fractured or are at risk of fracture due to osteoporosis need to avoid high-intensity exercise, such as high-intensity aerobic exercise, climbing stairs, skipping rope, etc. [42]. Guidelines for people with vertebral fractures recommend 20 min a day of balance training, posture awareness, strength training and aerobic training for each major muscle group at least twice a week [43].

The strengths of this study were the use of DXA transverse scanning of the thoracolumbar spine and the Optasia Spinalizer software to assess vertebral fractures using Genant's SQ technique, which is the standard for vertebral fracture assessment. It was recommended by the International Society for Clinical Densitometry [44]. The accuracy of vertebral fracture evaluation is ensured.

### Limitations

However, a few limitations were in our study. First, the cross-sectional design of this study makes it difficult to establish a causal association between calcium intake and MET and vertebral fractures. Second, MET and calcium intake in this study mainly reflected the situation at the time of the survey, but could not reflect the long-term calcium intake and exercise habits. The average value of calcium intake and supplements from two dietary surveys was used to reflect the calcium intake over a period of time in the study. Third, other factors that may influence fracture and fracture status at other sites were not considered in the data limited to the database. Therefore, prospective studies are needed to further investigate the effects of long-term physical activity and calcium intake on fracture risk at different sites and possible interactions.

## Conclusion

In this study, we found the combination of MET < 160 and calcium intake ≥ 670 mg may be associated with the decreased risks of vertebral fractures in participants over 50 years old. High-intensity exercise was not recommended for participants over 50 years old, but adequate calcium intake was recommended for them while taking low-intensity exercise.

## Supplementary Information

**Additional file 1: Supplementary Table 1.** Sensitivity analysis before and after deletion of missing values

## Data Availability

The datasets generated and/or analyzed during the current study are available in the NHANES database, https://wwwn.cdc.gov/nchs/nhanes/.

## Abbreviations

MET: Metabolic equivalent
NHANES: National Health and Nutrition Examination Surveys
BMI: Body mass index
BMD: Bone mineral density
DXA: Dual-energy x-ray absorptiometry
SQ: Semiquantitative
MEC: Mobile Examination Center
GED: General educational development
SD: Standard deviation
PSUs: Pseudo-primary sampling units
