# Supplementary material for: Role of metabolic equivalent between calcium intake and vertebral fractures: a cross-sectional study of NHANES 2013–2014
Source: BMC Geriatr. 2022 Dec 20;22:986. doi: 10.1186/s12877-022-03666-4 (PMC9768999; doi:10.1186/s12877-022-03666-4)
Supplement: Supplementary file 1 — Additional file 1: Supplementary Table 1. Sensitivity analysis before and after deletion of missing values [file 12877_2022_3666_MOESM1_ESM.docx]

**Supplementary Table 1**. Sensitivity analysis before and after deletion of missing values

|  | | Groups | |  | |
| --- | --- | --- | --- | --- | --- |
| Variables | Total (n=1675) | After deleting the missing value (n=766) | Before deleting the missing value (n=909) | Statistics | *P* |
| Age, years, Mean ± SD | 63.09 ± 8.24 | 63.10 ± 8.23 | 63.08 ± 8.25 | t=0.06 | 0.948 |
| Gender, n (%) |  |  |  | χ^2^=0.274 | 0.601 |
| Male | 1,001 (59.76) | 463 (60.44) | 538 (59.19) |  |  |
| Female | 674 (40.24) | 303 (39.56) | 371 (40.81) |  |  |
| Race, n (%) |  |  |  | χ^2^=0.479 | 0.489 |
| White | 949 (56.66) | 427 (55.74) | 522 (57.43) |  |  |
| Others (Mexican American, other Hispanic, non-Hispanic Black, and other races) | 726 (43.34) | 339 (44.26) | 387 (42.57) |  |  |
| Education level, n (%) |  |  |  | - | 0.953 |
| Less than 9th grade | 151 (9.01) | 65 (8.49) | 86 (9.46) |  |  |
| 9-11th grade (includes 12th grade with no diploma) | 218 (13.01) | 99 (12.92) | 119 (13.09) |  |  |
| High school graduate/GED or equivalent | 358 (21.37) | 161 (21.02) | 197 (21.67) |  |  |
| Some college or AA degree | 450 (26.87) | 208 (27.15) | 242 (26.62) |  |  |
| College graduate or above | 497 (29.67) | 233 (30.42) | 264 (29.04) |  |  |
| Unknown | 1 (0.06) | 0 (0.00) | 1 (0.11) |  |  |
| Family income to poverty ratio, M (Q_1_, Q_3_) | 2.43 (1.21, 5.00) | 2.46 (1.21, 5.00) | 2.41 (1.21, 4.84) | Z=0.450 | 0.652 |
| Height, cm, Mean ± SD | 167.82 ± 9.95 | 168.02 ± 9.81 | 167.66 ± 10.07 | t=0.74 | 0.456 |
| Weight, kg, Mean ± SD | 79.33 ± 18.49 | 79.13 ± 18.20 | 79.50 ± 18.74 | t=-0.41 | 0.682 |
| Waist circumference, cm, Mean ± SD | 99.52 ± 14.03 | 99.31 ± 13.95 | 99.70 ± 14.11 | t=-0.58 | 0.563 |
| BMI, kg/m^2^, Mean ± SD | 28.02 ± 5.47 | 27.90 ± 5.40 | 28.13 ± 5.53 | t=-0.85 | 0.394 |
| Smoking, n (%) |  |  |  | - | 0.874 |
| Yes | 808 (48.24) | 373 (48.69) | 435 (47.85) |  |  |
| No | 866 (51.70) | 393 (51.31) | 473 (52.04) |  |  |
| Unknown | 1 (0.06) | 0 (0.00) | 1 (0.11) |  |  |
| Drinking, n (%) |  |  |  | χ^2^=0.542 | 0.461 |
| Yes | 1231 (74.74) | 579 (75.59) | 652 (74.01) |  |  |
| No | 416 (25.26) | 187 (24.41) | 229 (25.99) |  |  |
| Diabetes, n (%) |  |  |  | χ^2^=0.168 | 0.682 |
| No | 1278 (76.30) | 588 (76.76) | 690 (75.91) |  |  |
| Yes | 397 (23.70) | 178 (23.24) | 219 (24.09) |  |  |
| Mother ever fracture a hip, n (%) |  |  |  | χ^2^=0.436 | 0.804 |
| Yes | 93 (5.55) | 40 (5.22) | 53 (5.83) |  |  |
| No | 1520 (90.75) | 699 (91.25) | 821 (90.32) |  |  |
| Unknown | 62 (3.70) | 27 (3.52) | 35 (3.85) |  |  |
| Father ever fracture a hip, n (%) |  |  |  | χ^2^=0.016 | 0.992 |
| Yes | 49 (2.93) | 22 (2.87) | 27 (2.97) |  |  |
| No | 1539 (91.88) | 704 (91.91) | 835 (91.86) |  |  |
| Unknown | 87 (5.19) | 40 (5.22) | 47 (5.17) |  |  |
| Broken or fractured hip, n (%) |  |  |  | χ^2^=0.031 | 0.861 |
| Yes | 21 (1.25) | 10 (1.31) | 11 (1.21) |  |  |
| No | 1654 (98.75) | 756 (98.69) | 898 (98.79) |  |  |
| Lipid-lowering agents, n (%) |  |  |  | χ^2^=0.053 | 0.817 |
| No | 1095 (65.37) | 503 (65.67) | 592 (65.13) |  |  |
| Yes | 580 (34.63) | 263 (34.33) | 317 (34.87) |  |  |
| Anti-osteoporosis therapy, n (%) |  |  |  | χ^2^=0.125 | 0.724 |
| No | 1649 (98.45) | 755 (98.56) | 894 (98.35) |  |  |
| Yes | 26 (1.55) | 11 (1.44) | 15 (1.65) |  |  |
| Glucocorticoids, n (%) |  |  |  | χ^2^=0.090 | 0.764 |
| No | 1644 (98.15) | 751 (98.04) | 893 (98.24) |  |  |
| Yes | 31 (1.85) | 15 (1.96) | 16 (1.76) |  |  |
| Total femur BMD, gm/cm^2^, M (Q_1_, Q_3_) | -0.02 (-0.71, 0.62) | -0.03 (-0.72, 0.63) | -0.02 (-0.71, 0.61) | Z=-0.041 | 0.967 |
| Femoral neck BMD, gm/cm^2^, M (Q_1_, Q_3_) | -0.07 (-0.71, 0.59) | -0.08 (-0.71, 0.61) | -0.07 (-0.70, 0.59) | Z=-0.035 | 0.972 |
| Osteopenia, n (%) |  |  |  | χ^2^=2.871 | 0.090 |
| Yes | 359 (21.43) | 150 (19.58) | 209 (22.99) |  |  |
| No | 1316 (78.57) | 616 (80.42) | 700 (77.01) |  |  |
| Energy, kcal, M (Q_1_, Q_3_) | 1862.00 (1429.50, 2391.50) | 1867.75 (1440.00, 2410.00) | 1858.50 (1424.50, 2382.00) | Z=0.671 | 0.502 |
| Vitamin D, mcg, M (Q_1_, Q_3_) | 8.55 (3.00, 22.90) | 8.43 (3.00, 22.45) | 8.60 (3.05, 22.90) | Z=-0.247 | 0.805 |
| Total calcium intake, mg, M (Q_1_, Q_3_) | 980.00 (667.00, 1436.00) | 981.00 (670.50, 1436.00) | 980.00 (664.50, 1426.50) | Z=0.198 | 0.843 |
| MET, M (Q_1_, Q_3_) | 240.00 (140.00, 480.00) | 280.00 (160.00, 480.00) | 240.00 (120.00, 480.00) | Z=0.975 | 0.330 |

SD: standard deviation, M: median, Q1: 1st quartile, Q3: 3st quartile; BMI: body mass index; MET: metabolic equivalent of energy; BMD: bone mineral density
